# Urinary N‐acetyl‐β‐d‐glucosaminidase‐creatine ratio is a valuable predictor for advanced diabetic kidney disease

**DOI:** 10.1002/jcla.24769

**Published:** 2022-12-26

**Authors:** Qinghua Huang, Xianming Fei, Huifang Zhan, Jianguang Gong, Jieru Zhou, Yulv Zhang, Xiao Ye, Yingxiang Song, Jiangbo Ma, Xiaohong Wu

**Affiliations:** ^1^ Suzhou Medical College of Soochow University Suzhou Jiangsu China; ^2^ Geriatric Medicine Center, Department of Endocrinology Zhejiang Provincial People's Hospital, Affiliated People's Hospital, Hangzhou Medical College Zhejiang China; ^3^ Key Laboratory of Endocrine Gland Diseases of Zhejiang Province Zhejiang China; ^4^ Laboratory Medicine Center, Department of Clinical Laboratory Zhejiang Provincial People's Hospital, Affiliated People's Hospital, Hangzhou Medical College Zhejiang China; ^5^ Department of Emergency Zhejiang University Hospital, the Second Affiliated Hospital of Medical College of Zhejiang University Hangzhou China; ^6^ Laboratory of Kidney Disease Zhejiang Provincial People's Hospital, Affiliated People's Hospital, Hangzhou Medical College Zhejiang China; ^7^ Jinzhou Medical University Jinzhou China

**Keywords:** Diabetic kidney disease, N‐acetyl‐β‐D‐glucosaminidase‐Creatinine ratio, Progression, Predictor

## Abstract

**Background:**

Many biomarkers show high diagnostic values for diabetic kidney disease (DKD), but fewer studies focus on the predictive assessment of DKD progression by blood and urinary biomarkers.

**Aim:**

This study aims to find powerful risk predictors and identifying biomarkers in blood and urine for DKD progression.

**Methods:**

A total of 117 patients with type 2 DKD including early and advanced stages and their laboratory parameters were statistically assessed. A receiver operating characteristic (ROC) curve analysis was performed to evaluate the significance of discriminating between early and advanced DKD, and the predictive power for advanced DKD was analyzed by regression analysis and trisector grouping.

**Results:**

N‐acetyl‐β‐d‐glucosaminidase‐creatine (NAG/CR) level in advanced DKD was statistically higher than that in early DKD (*p* < 0.05), and there was a higher incidence of advanced DKD (72% vs. 56%) and high odds ratio (OR: 3.917, 95% CI: 1.579–10.011) of NAG/CR with ≥2.79 U/mmol compared with <2.79 U/mmol (*p* < 0.05). NAG/CR ratio also showed a higher area under the ROC curve of 0.727 (95% CI: 0.616–0.828, *p* = 0.010) with a high sensitivity (0.75) and a moderate specificity (0.66) when 1.93 U/mmol was set as the optimal cutoff value. The adjusted‐multivariable analysis revealed that NAG/CR had an OR of 1.021 (95% CI: 1.024–1.038) and 2.223 (95% CI: 1.231–4.463) based on a continuous and categorical variable, respectively, for risk of advanced DKD. Moreover, the prevalence of advanced DKD exhibited an increasing tendency by an increment of the trisector of NAG/CR.

**Conclusions:**

This study suggests that NAG/CR ratio is an independent predictor for advanced DKD, and it also can be used as a powerful identifying marker between early and advanced DKD.

## Graphical abstract

Regression analysis revealed urinary NAG/CR ratio is an independent risk predictor for advanced DKD, and ROC curve analysis showed it is a powerful indicator for discriminating between early and advanced DKD.

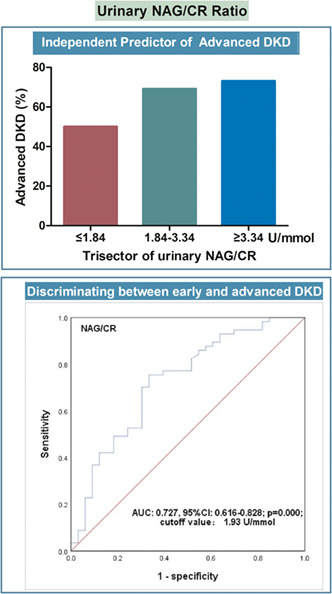

## 1 INTRODUCTION

Diabetes is the leading cause of chronic kidney disease and end‐stage kidney disease worldwide and becomes one of the biggest healthcare challenges of the 21st century.^1^ Microvascular complications have been identified as the common and major diabetic complications.^2^, ^3^ In which, diabetic kidney disease (DKD) is the main one leading factor to final kidney failure and death of patients.^4^ DKD typically manifests a progressive deterioration of kidney function, and the increased glomerular filtration rate (GFR), glomerular hypertrophy, and excretion of urinary albumin are the major indicators.^5^ Moreover, DKD is not only closely associated with poor outcomes of diabetic patients^6^ but also a powerful predictor of mortality in diabetic patients.^7^ Therefore, it would be of great importance to early predict and prevent DKD progression to decrease the mortality in diabetes.

The severity of DKD can also be assessed by clinical and pathological methods. DKD has traditionally graded into four stages in pathology according to the Renal Pathology Society classification,^8^ and both stages 3 and 4 are described as advanced DKD, but stage 4 represents the most severe condition, namely end‐stage renal disease (ESRD).^8^, ^9^ Thus, the right DKD class grading and early identification and diagnosis of advanced DKD especially stage 4 could be the key point of introducing preventive and treating interventions, which might contribute to improving the prognosis of DKD patients. In clinical practice, urinary albumin excretion (UAE) in 24 h and urine albumin to creatine ratio (UACR) are commonly recognized as good diagnostic, progressive, and predictive markers for DKD. However, urine microalbumin (UMA) lacks sufficient sensitivity and specificity to predict the progression of DKD.^10^, ^11^ Some previous studies have indicated that a lot of indicators and biomarkers were associated with microvascular damage and could reveal its presence in type 2 diabetes (T2D) patients,^12^, ^13^ and these parameters also proved to be related to the development and progression of T2D.^14^, ^15^ However, the associations of these indicators with risk and progression as well as different disease conditions are still unclear.

In the past two or three decades, the potential roles of some biomarkers in the progression and prognosis of diabetic nephropathy (newly named DKD) have been validated and confirmed,^16^ and the clinical significance of many biomarkers in the incidence and prevalence of DKD has been well recognized. Our latest studies indicated that serum thyroid stimulating hormone and free triiodothyronine,^17^ plasma fibrinogen,^18^ as well as the peripheral monocyte‐to‐lymphocyte ratio,^19^ were independent risk predictors of DKD, suggesting that routine laboratory indicators may probably be useful parameters for discriminating between early and advanced stages of DKD, and may be potential risk predictors for advanced DKD. Although microalbuminuria and other urine proteins proved to be good markers of early kidney injury, various urine proteins may exhibit significant differences in diagnostic, predictive, and prognostic power for DKD, which has not been completely confirmed in DKD patients diagnosed pathologically. Therefore, the main aim of the present study was to reveal the clinical significance of urinary protein measurements in pathological grading and risk assessment of advanced DKD.

## 2 MATERIALS AND METHODS

### 2.1 Patients population

A total of 117 patients with type 2 DKD confirmed by renal puncture pathology were included and were retrospectively analyzed according to the inclusion and exclusion criteria. The subjects were those in the Department of Endocrinology, Zhejiang Provincial People's Hospital between 2015 and 2021, including 83 males and 34 females, aged 37–85 years. The diagnosis of DKD acorded with the criteria in the diabetes guideline of the China Diabetes Association.^20^, ^21^, ^22^ The pathophysiological and clinical characteristics of the patients before treatment, including age, sex, duration of diabetes, carotid atherosclerosis (AS), systolic blood pressure (SBP), diastolic blood pressure (DBP), and body mass index (BMI) were collected from hospital information system and reviewed. The inclusion criteria included: (1) type 2 diabetes and (2) untreated diabetic nephropathy diagnosed by pathology. The exclusion criteria were as follows: (1) primary liver and kidney disorders; (2) recent history of surgery; (3) cardiovascular and cerebrovascular diseases; (4) cancers; and (5) acute infections. The patients were divided into two groups: early (stages IIa and IIb) and advanced (stages III and IV) groups based on the pathological grading. AS a retrospective review, the patients consent was waived, and the study has been approved by the ethical committee of Zhejiang Provincial People's Hospital (Approval No. 2021KT014).

### 2.2 Laboratory assay

The urine in spot time and whole 24 h was obtained, and the fasting venous blood of patients were collected in vacutainer tubes with sodium citrate (2.7 ml blood sample), EDTA‐K2 (2 ml blood sample), and anticoagulant‐free (5 ml blood sample) (BD) before treatment, respectively. An automatic hematological analyzer (BC‐7500; Mindray Inc) was used to analyze the white blood cell (WBC), neutrophil (NEU), and high sensitive C reactive protein (HS‐CRP). The serum was isolated by 1500*g*‐centrifugation at room temperature for 5 min. Subsequently, the levels of multiple biochemical indexes were measured, which included serum creatine (sCR), serum urea (sUR), cholesterol (CHOL), homocysteine (HCY), thyroid stimulating hormone (TSH), free triiodothyronine (FT3), free thyroxine (FT4), fibrinogen (FIB), and d‐dimer (D‐D). At the same time, the levels of urinary creatinine, N‐acetyl‐β‐d‐glucosaminidase (NAG), total protein (PRO), micoralbumin (MA), immunoglobulin G (IgG), transferin (TFR), and α1‐microglobulin (α1MG) in spot urine, and albumin excretion (UAE) in 24 h urine were measured by the fully biochemical analyzer (AU5800, Beckman‐coulter). Finally, urinary NAG and proteins to creatinine ratios were calculated to present as NAG/CR, PRO/CR, albumin to creatine ratio (ACR), IgG/CR, TFR/CR, and α1MG/CR. The estimated glomerular filtration rate (eGFR) was obtained by the formula: eGFR = 133× (CysC/0.8)^−0.449 (−1.328 if CysC >0.8mg/L)^ × 0.996^age^ × (0.932 if female).^23^ The minimum detection level and the intra‐ and inter‐coefficient of variation (CV) for each assay in this study were listed in Table 1.

**TABLE 1:** Basic performance parameters of all the assays in the study.

Parameters	Minimum detection level	Intra‐CV (%)	Inter‐CV (%)
HbA1c	15 mmol/mol	0.60	1.40
WBC	0.00 × 10^9^/L	4.00	6.00
NEU	0.00 × 10^9^/L	2.13*	4.00*
HS‐CRP	0.2 mg/L	1.61*	2.88*
sCR	4.4 μmol/L	1.20	3.50*
sUR	0.8 mmol/L	3.00	5.00*
CHOL	0.5 mmol/L	3.00	5.00*
HCY	2.5 μmol/L	3.00	5.41*
TSH	0.005 mIU/L	2.00	3.00
FT3	0.88 ng/L	6.60	8.00
FT4	0.3 μIU/ml	4.40	8.08
Fbg	0.80 g/L	5.00	10.00
D‐D	160 μg/L	15.00	15.00
MA	8.00 mg/L	5.00	8.00
IgG	4.00 mg/L	5.00	8.00
NAG	1.50 U/L	10.0	10.0
TRF	0.20 mg/L	5.00	8.00
α1MG	0.40 mg/L	5.00	8.00

*Note*: Data were referred to the instructions of the reagent kits. MA, IgG, NAG, TRF, and α1MG are the urine parameters.

Abbreviation: CV, coefficient of variation.

* The data were from the results of performance verification in the Laboratory Medicine Center of the hospital.

### 2.3 Statistical analysis

Initially, the Kolmogorov–Smirnov test was used to analyze the distribution normality of the data, and they would be presented as mean ± standard deviation and median when distributing normally and nonnormally, respectively. Mann–Whitney *U* test and Student's *t*‐test were used for the nonnormal and normal distribution data of the patient's characteristics, respectively. Categorical data (percent) were analyzed by Chi‐square test. The area under the receiver operating characteristic (ROC) curve was calculated to assess the identifying power for early and advanced DKD. Logistic regression analysis was carried out to calculate the odds ratio for advanced DKD. The statistical package SPSS 20.0 (SPSS, IBM Corp) was used in the study. A *p‐*Value of less than 0.05 (which was 0.10 for regression analysis) was considered statistically significant.

## 3 RESULTS

### 3.1 Characteristics and indicators in early and advanced DKD patients

The 117 DKD patients were assigned two groups including group early DKD (stage II, 27 with IIa and 19 with IIb), and the group advanced DKD (35 with stage III and 36 with stage IV). In all characteristics, only the duration of diabetes exhibited a significant difference between the two groups (*p* < 0.05). However, the levels of FT3, UAE, ACR, PRO/CR, IgG/CR, and NAG/CR were statistically significant (*p* < 0.05) in the measured biomarkers. The results demonstrated that patients with advanced DKD were more likely to have a longer duration of DKD, lower FT3 levels, and higher levels of ACR, PRO/CR, IgG/CR, and NAG/CR. The detailed data were presented in Table 2.

**TABLE 2:** Comparisons of basic characteristics and biomarkers between early and advanced DKD.

Variables	Early DKD (*n* = 46)	Advanced DKD (*n* = 71)	Statistical value	*p‐*Value
Sex (M/total, %)	34/46 (73.9)	48/71 (67.6)	0.530	0.467
Age (years)	53.3 ± 9.4	54.7 ± 11.4	0.707	0.498
Duration of diabetes (years)	9.2 (0.1–20.0)	10.6 (1.0–30.0)	984.00	0.017
Smoking (*n*/*n*, %)	8/43 (18.6)	19/71 (26.8)	0.986	0.321
BMI (kg/m^2^)	25.97 ± 3.41	25.28 ± 5.11	0.777	0.439
Carotid AS (*n*/*n*, %)	27/46 (58.7)	31/70 (44.3)	2.306	0.129
SBP (mmHg)	143.7 ± 23.4	149.8 ± 22.2	1.389	0.168
DBP (mmHg)	83.1 ± 12.7	80.3 ± 12.0	1.155	0.251
HbA1c (%)	7.55 (5.50–15.40)	6.95 (5.10–14.10)	1206.500	0.527
WBC(x 10^9^/L)	7.04 ± 2.29	6.87 ± 2.57	0.828	0.727
NLR	3.06 ± 2.04	3.30 ± 2.30	0.577	0.565
HS‐CRP (mg/L)	3.30 (1.30–53.6)	1.50 (0.4–51.3)	761.500	0.051
sCR (mmol/L)	148.9 (66.9–627.6)	152.2 (56.0–574.1)	1497.000	0.655
sUR (mmol/L)	9.26 (2.55–27.91)	9.97 (2.11–28.44)	1260.000	0.733
CHOL (mmol/L)	5.02 (2.37–12.6)	4.86 (2.21–13.76)	1501.500	0.674
HCY (μmol/L)	18.11 ± 5.58	17.72 ± 8.46	0.198	0.844
eGFR (ml/min)	44.3 ± 27.8	50.4 ± 27.8	0.876	0.384
TSH (U/L)	1.97 (0.02–30.78)	2.76 (0.56–95.09)	689.000	0.085
FT3 (ng/L)	2.90 ± 0.62	2.58 ± 0.47	2.023	0.046
FT4 (ng/dl)	8.44 ± 1.69	9.09 ± 1.76	0.799	0.427
FIB (g/L)	4.07 ± 1.54	4.26 ± 1.40	0.622	0.491
D‐D (μg/L)	570 (30–8410)	870 (80–4660)	823.500	0.018
UAE (mg/24 h)	1300 (61–6902)	2607 (198–21,300)	478.000	0.568
ACR (mg/g)	84.6 (7.0–772.0)	204.9 (1.0–1078.0)	532.000	0.021
PRO/CR (g/g)	1.65 (0.01–9.13)	3.87 (0.13–14.52)	687.500	0.017
IgG/CR (mg/mmol)	13.3 (0.64–224.3)	50.24 (1.5–413.3)	513.500	0.000
NAG/CR (U/mmol)	2.40 ± 1.53	4.01 ± 2.27	2.036	0.045
TRF/CR (mg/mmol)	11.2 (0.3–340.4)	26.0 (0.5–461.5)	582.500	0.491
α1MG/CR (mg/mmol)	7.2 (1.0–105.0)	10.9 (1.4–71.4)	715.500	0.091

*Note*: Data were presented as mean and standard deviation, the median and range, and percentage analyzed by Student *t*‐test, Mann–Whitney *U* test, and Chi‐square test, respectively.

### 3.2 Incidence and risk analysis of advanced DKD based on the thresholds

In the study, we observed the incidence of advanced DKD in different status and levels of variables. The observed variables included those with *p* values of less than 0.100 compared with pure T2D. When the cutoff values of continuous variables were set at the median or medically significant levels, the incidence of advanced DKD showed a remarkable difference in different levels of the duration of diabetes, ACR, PRO/CR, Ig/CR, and NAG/CR (*p* < 0.05), and NAG/CR showed a highest OR value of 3.917 (95% CI: 1.579–10.011). Detailed results were in Table 3.

**TABLE 3:** Incidence and univariate analysis of advanced DKD in different characteristics.

Comparison	Prevalence of advanced DKD (%)	OR (95% CI)	*p*‐Value
Sex, male vs. female	59 vs. 64	1.318 (0.576–3.012)	0.513
Age (years), ≥53 vs. <53	59 vs. 63	1.863 (0.741–4.680)	0.186
Duration of diabetes (years), ≥10 vs. <10	67 vs. 53	1.781 (1.211–3.941)	0.015*
Smoking habit, yes vs. no	70 vs. 58	0.576 (0.228–1.454)	0.243
BMI (kg/m^2^), ≥24 vs. <24	56 vs. 64	0.720 (0.325–1.594)	0.417
AS, yes vs. no	53 vs. 67	1.788 (0.842–3.796)	0.115
SBP (mmHg), ≥130 vs. <130	64 vs. 46	2.105 (0.845–5.241)	0.110
DBP (mmHg), ≥80 vs. <80	58 vs. 64	0.799 (0.362–1.677)	0.523
HbA1c (%), ≥7.0 vs. <7.0	55 vs. 56	0.980 (0.803–1.196)	0.845
HS‐CRP (mg/L), ≥2.0 vs. <2.0	42 vs. 72	0.971 (0.935–1.007)	0.113
FT3 (ng/L), <2.64 vs. ≥2.64	65 vs. 67	0.421 (0.176–1.004)	0.051*
D‐D (ng/ml), ≥700 vs. <700	71 vs. 51	0.913 (0.676–1.331)	0.637
ACR (mg/g), ≥300 vs. <300	74 vs. 52	2.676 (1.102–6.509)	0.030*
PRO/CR (g/g), ≥3.0 vs. <3.0	77 vs. 50	3.333 (1.298–8.557)	0.012*
IgG/CR (mg/mmol), ≥36.05 vs. <36.05	78 vs. 49	3.659 (1.467–9.127)	0.005*
NAG/CR (U/mmol), ≥2.79 vs. <2.79	72 vs. 56	3.937 (1.579–10.011)	0.002*
α1MG/CR (mg/mmol), ≥9.075 vs. <9.075	73 vs. 58	0.998 (0.973–1.023)	0.856

*Note*: The cutoff points of continuous variables were the median values, but that of SBP, DBP, HbA1c, ACR, and PRO/CR were the medically significant levels. *p*‐Value: analyzed by Chi‐square test.

Abbreviations: CI, confidence interval; OR, odds ratio.

* Indicating the *p*‐Values are of statistical significance.

### 3.3 ROC curve analysis for discriminating between early and advanced DKD

**FIGURE 1:**
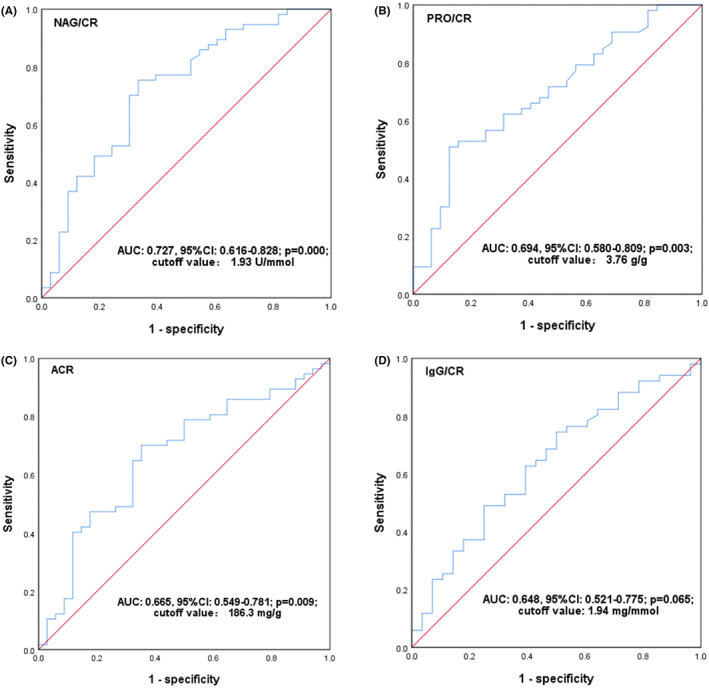
ROC curves for discriminating between early and advanced DKD. ROC curves marked with (A), (B), (C), and (D) represent NAG/CR, PRO/CR, ACR, and IgG/CR, respectively. AUC, area under curve; CI, confidence interval.

Using the data of duration of disease, ACR, PRO/CR, IgG/CR, and NAG/CR, ROC curve analysis was constructed to evaluate the value for discriminating between early and advanced DKD. The results demonstrated a higher AUC of 0.727 (95% CI: 0.616–0.828, *p* = 0.010) for the NAG/CR ratio than that of an AUC of less than 0.700 for other variables, respectively **(**Figure 1 **)**. When the optimal cutoff value was 1.93 U/mmol, NAG/CR exhibited a high sensitivity (0.75), a moderate specificity (0.66), and a positive and negative predictive value of 0.69 and 0.73, respectively.

### 3.4 Multivariate regression analysis of risk factors for advanced DKD

**FIGURE 2:**
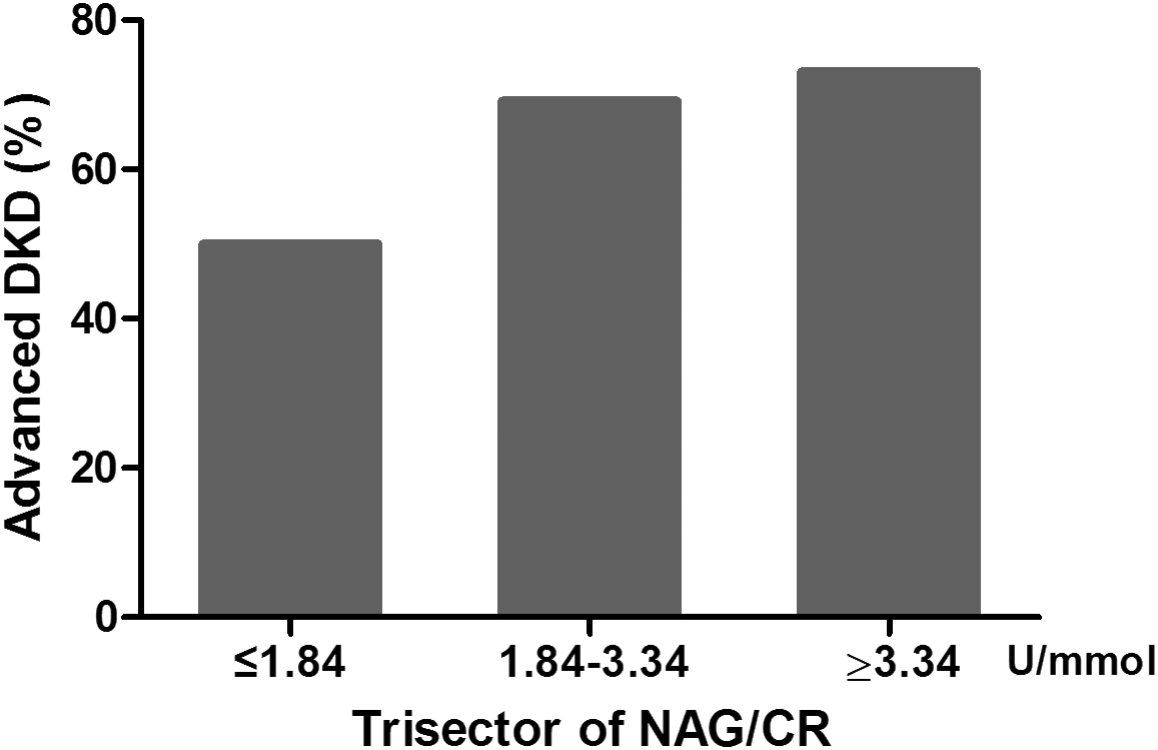
Prevalence of advanced DKD grouped by trisector of NAG/CR. The value of 1.84 and 3.34 U/mmol represents that of the first and second trisector (P_33_ and P_67_), respectively (*p*‐trend<0.05 analyzed by one‐way ANOVA).

According to the univariate and ROC curve analysis, the NAG/CR was treated as a continuous variable and categorical variable divided as trisectors and by the optimal cutoff value of 1.93 U/mmol, respectively. The adjusted‐multivariate analysis indicated that NAG/CR ratio had an OR of 1.021 (95% CI: 1.024–1.038) and 2.223 (95% CI: 1.231–4.463) when as a continuous variable and categorical variable, respectively, for progressive risk from early to advanced DKD (Table 4). Furthermore, when patients were assigned three groups based on the trisector of NAG/CR, the incidence of advanced DKD was 50.0%, 69.2%, and 73.1%, respectively (*p*‐trend<0.05), which indicates that the incidence of advanced DKD has an elevated trend with an increment of the trisectors (Figure 2).

**TABLE 4:** The adjusted multivariate logistic regression analysis of urinary NAG/CR for risk of advanced DKD.

NAG/CR level	Multivariate‐adjusted analysis	
	OR (95%CI)	*p*‐Value
Continuous variable	1.021 (1.004–1.038)	0.038*
Optimal cutoff from ROC curve		
<1.93 U/mmol	1.000	
≥1.93 U/mmol	2.223 (1.131–4.463)	0.010*

Abbreviation: ROC, receiver operating characteristic.

* Indicating the *p*‐Values are of statistical significance.

## 4 DISCUSSION

In pathology, the common manifestation of DKD is that of thickened glomerular basement membrane, expanded glomerular mesangial matrix, and forming glomerular nodular sclerosis in the advanced stages,^24^ and it is clinically described as proteinuria or declined renal function.^25^ In the different stages of DKD, patients may demonstrate different clinical features consistent with the severity of the disease. In DKD progression, some blood and urine biomarkers might directly reflect the disease condition and were proven to be good predictors.^26^, ^27^, ^28^, ^29^, ^30^ In the present study, our results showed that urinary NAG/CR levels exhibited remarkable difference between early and advanced DKD, and patient with advanced DKD was more likely to have higher levels of NAG/CR than that with early DKD. Our findings indicated that NAG/CR is closely associated with the severity of DKD, and may probably be a powerful risk predictor for advanced DKD, which suggests that the measurement of urinary NAG/CR level will be helpful to evaluate the condition and progression of DKD. Thus, it will be of great importance to investigate the clinical significance of the NAG/CR in discriminating between early and advanced DKD.

It is clear that urinary proteins and enzymes such as NAG from the kidney can better reveal kidney injury to some extent, but these markers have some deficiencies in sensitivity and specificity to detect kidney injury.^31^ Within the scope of our knowledge, the potential clinical values of urinary proteins and enzymes in predicting advanced DKD and discriminating between different severity of DKD is unclear. Therefore, we first assessed the incidence of advanced DKD in different status or levels of the observed markers. We found that there was a higher incidence of advanced DKD when ACR, PRO/CR, IgG/CR, and NAG/CR were above the median value or medically significant level, respectively. Univariable analysis according to the thresholds, ACR, PRO/CR, IgG/CR, and NAG/CR were all risk predictors for advanced DKD. However, NAG/CR had the highest OR value compared with other biomarkers, which revealed that NAG/CR ratio is a more powerful predictor than other proteins to creatinine ratios, and it can be used as a more clinically valuable predictor for advanced DKD.

NAG is a lysosomal enzyme, and a previous study has revealed that urinary NAG is probably a marker of increased lysosomal turnover when increased protein is expressed in the renal tubular cells.^32^ Therefore, NAG may be used as a good marker of DKD progression, and the combination of urinary NAG activity and creatine concentration will present a high power for the assessment of kidney injury in T2D patients by eliminating the influence of urine volume. Therefore, to reveal whether NAG/CR ratio was an independent risk factor of the progression of DKD, we further used the ROC curve combining adjusted‐multivariable regression analysis to assess the correlation of NAG/CR ratio with advanced DKD, and observed the significance of discriminating between early and advanced DKD. The study indicated that NAG/CR ratio had a higher area under curve of 0.727 than any of the urinary proteins to creatine ratios in discriminating between early and advanced DKD. Furthermore, based on an optimal cutoff value of 1.93 U/mmol, NAG/CR ratio also exhibited high sensitivity and moderate specificity, which suggests that NAG/CR ratio has a high identifying power for advanced DKD. In consideration of the influence of other confounding risk factors, adjusted‐multivariate analysis was performed based on the optimal cutoff value to observe whether NAG/CR was an independent predictor for advanced DKD. Our results demonstrated that urinary NAG/CR ratio was independently correlated with the risk of advanced DKD, and also exhibited that subjects with high trisector of NAG/CR ratio were likely to have a high incidence of advanced DKD, revealing that increasing NAG/CR ratio will be a powerful risk predictor of advanced DKD. Therefore, our results imply that NAG/CR ratio is an independent risk factor with a higher predictive power for advanced DKD, and it can be used as an independent predictor based on the optimal cutoff value.

There were at least three limitations in the present study. Firstly, the study probably did not exclude these DKD patients with other microvascular complications being not diagnosed because they might not have had typical clinical features before treatment. Therefore, the data of these patients might have produced some untrue results and could have influenced the predictive and identifying power. Secondly, the population lacking DKD with stage I. Therefore, it was uncertain whether there was an obvious difference in the NAG/CR ratio between different stages of early DKD. Thirdly, because of being difficult to obtain pathological samples of renal, the study had not enough DKD subjects in stages II and III, and there were also not enough patients for the validation of results, which probably leaded an uncertain conclusion. Despite the limitations, our study also revealed that the urinary NAG/CR ratio has a high predictive power for DKD progression from early to advanced stage diagnosed by pathology.

## 5 CONCLUSIONS

This study suggests that NAG/CR ratio is an independent predictor for advanced DKD in T2D patients, and it also can be used as a powerful identifying marker between early and advanced DKD. However, in consideration of some probable limitations in study design, subject collection, and sample size, further prospective studies with rigorous design and large samples of subjects may provide more definitive results.

## FUNDING INFORMATION

This work was supported by the Public Welfare Technology Application Research Project of Zhejiang Province, China (Grant number LGD20H070002, LGD21H020004), the Research Project of Zhejiang Provincial People's Hospital (Grant number ZRY2020B014), the Medicine and Health Science and Technology Project of Zhejiang Province (Grant numbers 2022KY523, 2020KY022, and 2021KY060), and the Traditional Chinese Medicine Research Project of Zhejiang Province, China (Grant number 2019ZB010). All the funders were not involved in the manuscript writing, editing, approval, or decision to publish.

## CONFLICT OF INTEREST

There was no conflict of interest.

## Data Availability

The data used to support the findings of this study are available from the corresponding author upon request.
